# *catena*-Poly[[bis­(μ_2_-4-amino­benzene­sulfonato-κ^2^ *O*:*O*)disilver]-bis­(μ_2_-4,4′-bipyridine-κ^2^ *N*:*N*′)]

**DOI:** 10.1107/S160053680803804X

**Published:** 2008-11-22

**Authors:** Guang-Chuan Ou, Min Zhang, Xian-You Yuan, Yong-Qiang Dai

**Affiliations:** a Department of Biology and Chemistry, Hunan University of Science and Engineering, Yongzhou, Hunan 425100, People’s Republic of China

## Abstract

In the title compound, [Ag_2_(C_6_H_6_NO_3_S)_2_(C_10_H_8_N_2_)_2_]_*n*_, the Ag^I^ atom is four-coordinated by two N atoms from two symmetry-related 4,4′-bipyridine (bipy) and two O atoms from two independent 4-amino­benzene­sulfonate (ABS) ligands. The two inter-chain Ag^I^ atoms are bridged by two independent ABS ligands through weak Ag—O bonds and Ag⋯Ag attractions, forming a ladder-like chain coordination polymer [Ag_2_(ABS)_2_(bipy)_2_]_*n*_ parallel to [001], which is further linked to generate a two-dimensional structure *via* N—H⋯O hydrogen-bonding inter­actions.

## Related literature

For general background, see: Liu, Kuroda-Sowa *et al.* (2005 ▶); Liu, Liu *et al.* (2005 ▶); Feng *et al.* (2003 ▶); Wei *et al.* (2004 ▶); Dong *et al.* (2005 ▶); Bi *et al.* (2003 ▶); Ding *et al.* (2005 ▶); Yang *et al.* (2004 ▶). For related structures, see: Sampanthar & Vittal (2000 ▶); Tong *et al.* (2000 ▶).
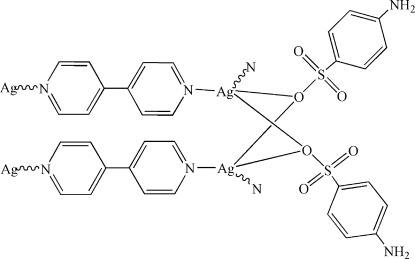


## Experimental

### 

#### Crystal data

[Ag_2_(C_6_H_6_NO_3_S)_2_(C_10_H_8_N_2_)_2_]
*M* *_r_* = 872.46
Monoclinic, 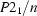

*a* = 9.2105 (19) Å
*b* = 15.774 (3) Å
*c* = 11.433 (2) Å
β = 108.004 (4)°
*V* = 1579.8 (6) Å^3^
*Z* = 2
Mo *K*α radiation
μ = 1.43 mm^−1^
*T* = 173 (2) K
0.42 × 0.13 × 0.12 mm

#### Data collection

Bruker SMART CCD area-detector diffractometer
Absorption correction: multi-scan (*SADABS*; Sheldrick, 1996 ▶) *T*_min_ = 0.585, *T*_max_ = 0.847
7741 measured reflections
3375 independent reflections
2774 reflections with *I* > 2σ(*I*)
*R*_int_ = 0.023

#### Refinement

*R*[*F* ^2^ > 2σ(*F* ^2^)] = 0.032
*wR*(*F* ^2^) = 0.088
*S* = 1.11
3375 reflections
217 parameters
H-atom parameters constrained
Δρ_max_ = 0.79 e Å^−3^
Δρ_min_ = −0.69 e Å^−3^

### 

Data collection: *SMART* (Bruker, 1997 ▶); cell refinement: *SAINT* (Bruker, 2003 ▶); data reduction: *SAINT*; program(s) used to solve structure: *SHELXS97* (Sheldrick, 2008 ▶); program(s) used to refine structure: *SHELXL97* (Sheldrick, 2008 ▶); molecular graphics: *DIAMOND* (Brandenburg, 2005 ▶); software used to prepare material for publication: *SHELXL97*.

## Supplementary Material

Crystal structure: contains datablocks I, global. DOI: 10.1107/S160053680803804X/pv2110sup1.cif

Structure factors: contains datablocks I. DOI: 10.1107/S160053680803804X/pv2110Isup2.hkl

Additional supplementary materials:  crystallographic information; 3D view; checkCIF report

## Figures and Tables

**Table 1:** Hydrogen-bond geometry (Å, °)

*D*—H⋯*A*	*D*—H	H⋯*A*	*D*⋯*A*	*D*—H⋯*A*
N3—H3*B*⋯O2^i^	0.88	2.04	2.850 (5)	153
N3—H3*C*⋯O3^ii^	0.88	2.25	2.905 (4)	131

Symmetry codes: (i) 
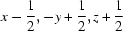
; (ii) 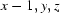.

**Fig. 1.**
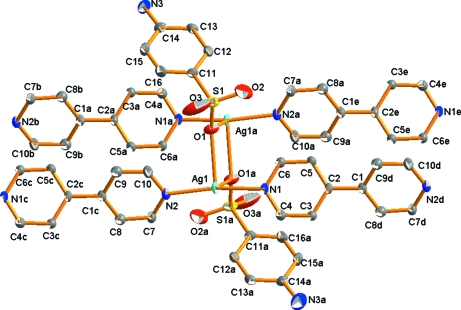
The molecular structure of the title compound, showing displacement ellipsoids at the 30% probability level; H-atoms have been excluded for clarity. The symmetry codes for the generated atoms: a (1 - x, -y, 1 - z), b (1 - x, -y, 2 - z), c (x, y, 1 + z), d (x, y, -1 + z), e (1 - x, -y, -z).

**Fig. 2.**
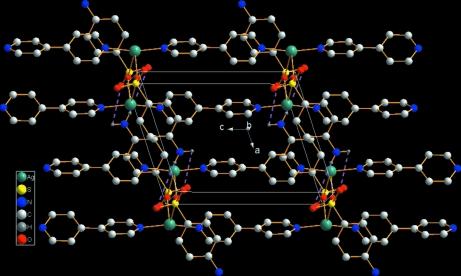
A view of the packing of the title compound along b axis.

## supplementary crystallographic information

### Comment

In the construction of inorganic–organic supramolecular complexes, the Ag^I^ is often a favorable candidate due to its flexible coordination modes and Ag–Ag attractions (Liu *et al.*, 2005; Dong *et al.*, 2005; Bi *et al.*, 2003; Ding *et al.*, 2005; Yang *et al.*, 2004). Bipy (4,4'-bipyridine) and ABS (4-aminobenzenesulfonic acid) are useful building blocks because they contain bifunctional groups, which can coordinate with metal ions in various coordination modes through the oxygen atoms of sulfonic group and the nitrogen atoms of pyridyl ring (Liu *et al.*, 2005; Feng *et al.*, 2003; Wei *et al.*, 2004). Therefore, we also extended these investigations to the use of the ligand ABS and obtained various framework structures. In this paper, we report the structure of the title compound, (I).

As illustrated in Fig. 1, each Ag^I^ atom in the title compound is four-coordinated by two nitrogen atoms from bipy (Ag1—N1 = 2.187 (3) Å, Ag1—N2 = 2.179 (3) Å) and two oxygen atoms from two independent ABS (Ag1—O1 = 2.572 (2) Å and Ag1—O1# = 2.654 (2) Å, # 1 - *x*, -*y*, 1 - *z*). These coordination modes are different from those found in structures similar to (I), wherein both oxygen atoms of acetic acid are linked to Ag atoms (Sampanthar & Vittal, 2000; Tong *et al.*, 2000). The two inter-chain Ag^I^ atoms are bridged by two independent ABS ligands through week Ag—O bonds and Ag–Ag attractions (Ag1–Ag1# = 3.903 Å, # 1 - *x*, -*y*, 1 - *z*), forming a one-dimensional ladder-like chain coordination polymer [Ag_2_(bipy)_2_(ABS)_2_]_n_ with periodical distance of 11.43 Å, which is further linked to generate a two-dimensional structure *via* hydrogen-bonding interactions with an average O–O distance of 2.877 Å (Fig. 2).

### Experimental

To a mixture of bipy (0.032 g, 0.2 mmol), ABS (0.017 g, 0.1 mmol) and Ag_2_O (0.028 g, 0.05 mmol) in CH_3_OH (10 ml) was added ammonia water resulting in a clear solution. After heating at 323 K for 0.5 h, the solution was evaporated slowly in the dark. Five days later, slightly yellow crystals were formed from the solution.

### Refinement

H atoms bound to C or N atoms were positioned geometrically and refined using the riding model, and with C—H = 0.95 Å and N—H = 0.88 Å, and with *U*(H) set to 1.2*U*_eq_(C, N). A small degree of thermal disorder in O2 and O3 atoms could not be ruled out as reflected by large atomic displacement parameters of these atoms.

### Crystal data

**Table:**

[Ag_2_(C_6_H_6_NO_3_S)_2_(C_10_H_8_N_2_)_2_]	*F*_000_ = 872
*M**_r_* = 872.46	*D*_x_ = 1.834 Mg m^−^^3^
Monoclinic, *P*2_1_/*n*	Mo *K*α radiation λ = 0.71073 Å
Hall symbol: -P 2yn	Cell parameters from 2489 reflections
*a* = 9.2105 (19) Å	θ = 2.5–27.0º
*b* = 15.774 (3) Å	µ = 1.43 mm^−^^1^
*c* = 11.433 (2) Å	*T* = 173 (2) K
β = 108.004 (4)º	Prism, light-yellow
*V* = 1579.8 (6) Å^3^	0.42 × 0.13 × 0.12 mm
*Z* = 2	

### Data collection

**Table:**

Bruker SMART CCD area-detector diffractometer	3375 independent reflections
Radiation source: fine-focus sealed tube	2774 reflections with *I* > 2σ(*I*)
Monochromator: graphite	*R*_int_ = 0.023
*T* = 173(2) K	θ_max_ = 27.0º
φ and ω scans	θ_min_ = 2.3º
Absorption correction: multi-scan(SADABS; Sheldrick, 1996)	*h* = −11→11
*T*_min_ = 0.585, *T*_max_ = 0.847	*k* = −17→20
7741 measured reflections	*l* = −13→14

### Refinement

**Table:**

Refinement on *F*^2^	Secondary atom site location: difference Fourier map
Least-squares matrix: full	Hydrogen site location: inferred from neighbouring sites
*R*[*F*^2^ > 2σ(*F*^2^)] = 0.032	H-atom parameters constrained
*wR*(*F*^2^) = 0.088	*w* = 1/[σ^2^(*F*_o_^2^) + (0.0458*P*)^2^ + 0.7557*P*] where *P* = (*F*_o_^2^ + 2*F*_c_^2^)/3
*S* = 1.11	(Δ/σ)_max_ = 0.001
3375 reflections	Δρ_max_ = 0.79 e Å^−^^3^
217 parameters	Δρ_min_ = −0.69 e Å^−^^3^
Primary atom site location: structure-invariant direct methods	Extinction correction: none

### Special details

**Table:**

Geometry. All e.s.d.'s (except the e.s.d. in the dihedral angle between two l.s. planes) are estimated using the full covariance matrix. The cell e.s.d.'s are taken into account individually in the estimation of e.s.d.'s in distances, angles and torsion angles; correlations between e.s.d.'s in cell parameters are only used when they are defined by crystal symmetry. An approximate (isotropic) treatment of cell e.s.d.'s is used for estimating e.s.d.'s involving l.s. planes.
Refinement. Refinement of *F*^2^ against ALL reflections. The weighted *R*-factor *wR* and goodness of fit *S* are based on *F*^2^, conventional *R*-factors *R* are based on *F*, with *F* set to zero for negative *F*^2^. The threshold expression of *F*^2^ > σ(*F*^2^) is used only for calculating *R*-factors(gt) *etc*. and is not relevant to the choice of reflections for refinement. *R*-factors based on *F*^2^ are statistically about twice as large as those based on *F*, and *R*- factors based on ALL data will be even larger.

### Fractional atomic coordinates and isotropic or equivalent isotropic displacement parameters (Å^2^)

**Table:**

	*x*	*y*	*z*	*U*_iso_*/*U*_eq_	
Ag1	0.72113 (3)	0.011584 (16)	0.56851 (2)	0.02788 (10)	
S1	0.43413 (9)	0.19537 (5)	0.48987 (9)	0.0333 (2)	
N1	0.7468 (3)	0.01972 (15)	0.3849 (2)	0.0216 (5)	
N2	0.7473 (3)	0.01021 (16)	0.7646 (2)	0.0250 (6)	
C2	0.7508 (3)	0.01617 (18)	0.1402 (3)	0.0203 (6)	
C12	0.1224 (3)	0.19650 (18)	0.4380 (3)	0.0236 (6)	
H12A	0.1175	0.1865	0.3549	0.028*	
C14	−0.0062 (3)	0.2155 (2)	0.5922 (3)	0.0274 (7)	
C1	0.7521 (3)	0.01415 (18)	0.0111 (3)	0.0211 (6)	
O1	0.4856 (2)	0.10869 (15)	0.5186 (2)	0.0362 (6)	
C11	0.2636 (3)	0.20524 (19)	0.5282 (3)	0.0228 (6)	
C15	0.1367 (4)	0.2258 (2)	0.6808 (3)	0.0299 (7)	
H15A	0.1427	0.2368	0.7638	0.036*	
C10	0.6969 (4)	0.0780 (2)	0.8123 (3)	0.0282 (7)	
H10A	0.6607	0.1256	0.7607	0.034*	
C7	0.8025 (4)	−0.0552 (2)	0.8414 (3)	0.0273 (7)	
H7A	0.8388	−0.1036	0.8097	0.033*	
C16	0.2681 (4)	0.2204 (2)	0.6487 (3)	0.0288 (7)	
H16A	0.3641	0.2270	0.7103	0.035*	
C13	−0.0112 (3)	0.2024 (2)	0.4698 (3)	0.0258 (7)	
H13A	−0.1072	0.1974	0.4078	0.031*	
C3	0.8647 (3)	−0.02265 (19)	0.2346 (3)	0.0240 (6)	
H3A	0.9463	−0.0514	0.2171	0.029*	
C6	0.6367 (3)	0.0573 (2)	0.2940 (3)	0.0249 (6)	
H6A	0.5563	0.0855	0.3141	0.030*	
C5	0.6351 (3)	0.0569 (2)	0.1740 (3)	0.0258 (7)	
H5A	0.5547	0.0846	0.1132	0.031*	
C4	0.8586 (4)	−0.01927 (19)	0.3527 (3)	0.0261 (7)	
H4A	0.9379	−0.0460	0.4155	0.031*	
O2	0.3940 (4)	0.2091 (3)	0.3591 (3)	0.0931 (15)	
N3	−0.1373 (3)	0.2149 (2)	0.6265 (3)	0.0483 (9)	
H3B	−0.1314	0.2211	0.7043	0.058*	
H3C	−0.2269	0.2084	0.5705	0.058*	
O3	0.5398 (3)	0.25465 (19)	0.5646 (4)	0.0858 (13)	
C8	0.8091 (4)	−0.0555 (2)	0.9632 (3)	0.0260 (7)	
H8	0.8518	−0.1024	1.0143	0.031*	
C9	0.6953 (4)	0.0818 (2)	0.9318 (3)	0.0277 (7)	
H9	0.6555	0.1304	0.9604	0.033*	

### Atomic displacement parameters (Å^2^)

**Table:**

	*U*^11^	*U*^22^	*U*^33^	*U*^12^	*U*^13^	*U*^23^
Ag1	0.03363 (16)	0.03836 (16)	0.01360 (14)	0.00271 (10)	0.01013 (10)	0.00153 (10)
S1	0.0248 (4)	0.0333 (5)	0.0465 (5)	0.0039 (3)	0.0178 (4)	0.0092 (4)
N1	0.0276 (13)	0.0222 (13)	0.0164 (12)	−0.0020 (10)	0.0090 (10)	−0.0024 (10)
N2	0.0300 (14)	0.0297 (14)	0.0160 (13)	0.0008 (11)	0.0082 (11)	0.0003 (11)
C2	0.0287 (15)	0.0198 (14)	0.0139 (14)	−0.0011 (12)	0.0085 (12)	−0.0014 (11)
C12	0.0287 (16)	0.0210 (15)	0.0198 (15)	0.0019 (12)	0.0054 (13)	0.0011 (12)
C14	0.0216 (15)	0.0269 (16)	0.0351 (19)	0.0021 (12)	0.0109 (14)	−0.0026 (14)
C1	0.0267 (15)	0.0211 (14)	0.0153 (14)	−0.0017 (12)	0.0060 (12)	−0.0013 (11)
O1	0.0277 (12)	0.0297 (13)	0.0522 (16)	0.0044 (9)	0.0138 (11)	−0.0036 (11)
C11	0.0188 (14)	0.0234 (15)	0.0272 (16)	0.0013 (11)	0.0086 (12)	0.0005 (13)
C15	0.0338 (17)	0.0356 (18)	0.0223 (17)	−0.0021 (14)	0.0115 (14)	−0.0086 (14)
C10	0.0416 (18)	0.0236 (16)	0.0194 (16)	0.0030 (13)	0.0094 (14)	0.0033 (13)
C7	0.0338 (17)	0.0296 (17)	0.0196 (16)	0.0051 (13)	0.0099 (13)	−0.0007 (13)
C16	0.0258 (16)	0.0323 (18)	0.0235 (17)	−0.0009 (13)	0.0005 (13)	−0.0066 (13)
C13	0.0208 (15)	0.0274 (16)	0.0251 (16)	0.0004 (12)	0.0010 (13)	0.0013 (13)
C3	0.0248 (15)	0.0269 (16)	0.0198 (16)	0.0053 (12)	0.0062 (12)	−0.0004 (12)
C6	0.0266 (15)	0.0278 (16)	0.0200 (15)	0.0046 (12)	0.0071 (12)	−0.0025 (13)
C5	0.0257 (15)	0.0335 (18)	0.0175 (15)	0.0061 (13)	0.0054 (12)	0.0002 (13)
C4	0.0288 (16)	0.0270 (16)	0.0208 (16)	0.0046 (13)	0.0053 (13)	−0.0017 (13)
O2	0.064 (2)	0.163 (4)	0.070 (2)	0.054 (2)	0.0474 (19)	0.071 (3)
N3	0.0304 (16)	0.080 (2)	0.0402 (19)	0.0081 (16)	0.0189 (14)	−0.0024 (18)
O3	0.0339 (15)	0.0463 (18)	0.185 (4)	−0.0175 (14)	0.045 (2)	−0.046 (2)
C8	0.0345 (17)	0.0243 (16)	0.0193 (15)	0.0075 (13)	0.0085 (13)	0.0024 (13)
C9	0.0390 (18)	0.0246 (16)	0.0210 (16)	0.0068 (13)	0.0114 (13)	0.0010 (13)

### Geometric parameters (Å, °)

**Table:**

Ag1—N2	2.179 (3)	C11—C16	1.387 (4)
Ag1—N1	2.187 (3)	C15—C16	1.372 (5)
Ag1—O1	2.571 (2)	C15—H15A	0.9500
S1—O3	1.428 (3)	C10—C9	1.372 (4)
S1—O2	1.441 (3)	C10—H10A	0.9500
S1—O1	1.451 (2)	C7—C8	1.375 (4)
S1—C11	1.763 (3)	C7—H7A	0.9500
N1—C4	1.345 (4)	C16—H16A	0.9500
N1—C6	1.345 (4)	C13—H13A	0.9500
N2—C10	1.347 (4)	C3—C4	1.370 (5)
N2—C7	1.348 (4)	C3—H3A	0.9500
C2—C3	1.393 (4)	C6—C5	1.368 (4)
C2—C5	1.398 (4)	C6—H6A	0.9500
C2—C1	1.480 (4)	C5—H5A	0.9500
C12—C13	1.390 (4)	C4—H4A	0.9500
C12—C11	1.394 (4)	N3—H3B	0.8800
C12—H12A	0.9500	N3—H3C	0.8800
C14—N3	1.380 (4)	C8—C1^ii^	1.399 (4)
C14—C15	1.400 (4)	C8—H8	0.9500
C14—C13	1.401 (4)	C9—C1^ii^	1.393 (4)
C1—C9^i^	1.393 (4)	C9—H9	0.9500
C1—C8^i^	1.399 (4)		
			
N2—Ag1—N1	167.73 (10)	N2—C10—C9	123.4 (3)
N2—Ag1—O1	92.98 (9)	N2—C10—H10A	118.3
N1—Ag1—O1	94.93 (9)	C9—C10—H10A	118.3
O3—S1—O2	115.6 (2)	N2—C7—C8	123.5 (3)
O3—S1—O1	111.53 (19)	N2—C7—H7A	118.2
O2—S1—O1	109.7 (2)	C8—C7—H7A	118.2
O3—S1—C11	106.77 (17)	C15—C16—C11	121.2 (3)
O2—S1—C11	106.38 (16)	C15—C16—H16A	119.4
O1—S1—C11	106.33 (14)	C11—C16—H16A	119.4
C4—N1—C6	116.7 (3)	C12—C13—C14	120.8 (3)
C4—N1—Ag1	123.7 (2)	C12—C13—H13A	119.6
C6—N1—Ag1	119.2 (2)	C14—C13—H13A	119.6
C10—N2—C7	116.8 (3)	C4—C3—C2	119.8 (3)
C10—N2—Ag1	117.8 (2)	C4—C3—H3A	120.1
C7—N2—Ag1	125.3 (2)	C2—C3—H3A	120.1
C3—C2—C5	116.4 (3)	N1—C6—C5	123.0 (3)
C3—C2—C1	121.8 (3)	N1—C6—H6A	118.5
C5—C2—C1	121.8 (3)	C5—C6—H6A	118.5
C13—C12—C11	119.9 (3)	C6—C5—C2	120.4 (3)
C13—C12—H12A	120.0	C6—C5—H5A	119.8
C11—C12—H12A	120.0	C2—C5—H5A	119.8
N3—C14—C15	120.3 (3)	N1—C4—C3	123.8 (3)
N3—C14—C13	121.4 (3)	N1—C4—H4A	118.1
C15—C14—C13	118.2 (3)	C3—C4—H4A	118.1
C9^i^—C1—C8^i^	117.2 (3)	C14—N3—H3B	120.0
C9^i^—C1—C2	121.0 (3)	C14—N3—H3C	120.0
C8^i^—C1—C2	121.8 (3)	H3B—N3—H3C	120.0
S1—O1—Ag1	143.69 (14)	C7—C8—C1^ii^	119.3 (3)
C16—C11—C12	119.1 (3)	C7—C8—H8	120.4
C16—C11—S1	120.5 (2)	C1^ii^—C8—H8	120.4
C12—C11—S1	120.4 (2)	C10—C9—C1^ii^	119.8 (3)
C16—C15—C14	120.6 (3)	C10—C9—H9	120.1
C16—C15—H15A	119.7	C1^ii^—C9—H9	120.1
C14—C15—H15A	119.7		
			
N2—Ag1—N1—C4	49.1 (6)	N3—C14—C15—C16	175.3 (3)
O1—Ag1—N1—C4	179.0 (2)	C13—C14—C15—C16	−2.2 (5)
N2—Ag1—N1—C6	−138.8 (4)	C7—N2—C10—C9	−2.1 (5)
O1—Ag1—N1—C6	−8.9 (2)	Ag1—N2—C10—C9	174.4 (3)
N1—Ag1—N2—C10	102.1 (5)	C10—N2—C7—C8	0.1 (5)
O1—Ag1—N2—C10	−28.0 (2)	Ag1—N2—C7—C8	−176.1 (2)
N1—Ag1—N2—C7	−81.8 (5)	C14—C15—C16—C11	0.6 (5)
O1—Ag1—N2—C7	148.2 (3)	C12—C11—C16—C15	0.8 (5)
C3—C2—C1—C9^i^	−147.0 (3)	S1—C11—C16—C15	−178.2 (3)
C5—C2—C1—C9^i^	33.1 (4)	C11—C12—C13—C14	−1.2 (5)
C3—C2—C1—C8^i^	33.5 (4)	N3—C14—C13—C12	−174.9 (3)
C5—C2—C1—C8^i^	−146.4 (3)	C15—C14—C13—C12	2.5 (5)
O3—S1—O1—Ag1	−41.8 (3)	C5—C2—C3—C4	0.0 (4)
O2—S1—O1—Ag1	87.6 (3)	C1—C2—C3—C4	−179.9 (3)
C11—S1—O1—Ag1	−157.8 (2)	C4—N1—C6—C5	0.1 (4)
N2—Ag1—O1—S1	102.2 (3)	Ag1—N1—C6—C5	−172.5 (2)
N1—Ag1—O1—S1	−68.4 (3)	N1—C6—C5—C2	0.1 (5)
C13—C12—C11—C16	−0.5 (5)	C3—C2—C5—C6	−0.2 (4)
C13—C12—C11—S1	178.4 (2)	C1—C2—C5—C6	179.7 (3)
O3—S1—C11—C16	−37.0 (3)	C6—N1—C4—C3	−0.3 (5)
O2—S1—C11—C16	−160.9 (3)	Ag1—N1—C4—C3	172.0 (2)
O1—S1—C11—C16	82.2 (3)	C2—C3—C4—N1	0.3 (5)
O3—S1—C11—C12	144.1 (3)	N2—C7—C8—C1^ii^	1.9 (5)
O2—S1—C11—C12	20.2 (3)	N2—C10—C9—C1^ii^	2.0 (5)
O1—S1—C11—C12	−96.7 (3)		

Symmetry codes: (i) *x*, *y*, *z*−1; (ii) *x*, *y*, *z*+1.

### Hydrogen-bond geometry (Å, °)

**Table:**

*D*—H···*A*	*D*—H	H···*A*	*D*···*A*	*D*—H···*A*
N3—H3B···O2^iii^	0.88	2.04	2.850 (5)	153
N3—H3C···O3^iv^	0.88	2.25	2.905 (4)	131

Symmetry codes: (iii) *x*−1/2, −*y*+1/2, *z*+1/2; (iv) *x*−1, *y*, *z*.
